# Exploring masculinities, sexual health and wellbeing across areas of high deprivation in Scotland: The depth of the challenge to improve understandings and practices

**DOI:** 10.1016/j.healthplace.2017.12.002

**Published:** 2018-03

**Authors:** Karen Lorimer, Lesley McMillan, Lisa McDaid, Dona Milne, Siân Russell, Kate Hunt

**Affiliations:** a Glasgow Caledonian University, School of Health&Life Sciences, Glasgow G4 0BA, Scotland, United Kingdom; b Glasgow Caledonian University, Glasgow School for Business&Society, Glasgow G4 0BA, Scotland, United Kingdom; c MRC/CSO Social&Public Health Sciences Unit, University of Glasgow, 200 Renfield Street, Glasgow G2 3QB, Scotland, United Kingdom; d NHS Lothian, Waverley Gate, 2-4 Waterloo Place, Edinburgh EH1 3EG, United Kingdom

**Keywords:** Masculinities, Sexual health, Area depriviation, Gender, Violence

## Abstract

Within and across areas of high deprivation, we explored constructions of masculinity in relation to sexual health and wellbeing, in what we believe to be the first UK study to take this approach. Our sample of 116 heterosexual men and women age 18–40 years took part in individual semi-structured interviews (n = 35) and focus group discussions (n = 18), across areas in Scotland. Drawing on a socio-ecological framework, findings revealed experience *in* places matter, with gender practices rooted in a domestically violent milieu, where localised, socio-cultural influences offered limited opportunities for more egalitarian performances of masculinity. We discuss the depths of the challenge in transforming masculinities in relation to sexual health and wellbeing in such communities.

## Highlights

• Gender practices were commonly rooted in a domestically violent milieu.
• Localised, socio-cultural influences offered limited opportunities for an egalitarian masculinity.
• Interventions need to operate across multiple levels and engage with multiple agencies.

## 1 Introduction

In a widely-cited, although not officially endorsed, definition from the World Health Organisation (WHO), sexual health is:

“…a state of physical, emotional, mental and social well-being in relation to sexuality; it is not merely the absence of disease, dysfunction or infirmity. Sexual health requires a positive and respectful approach to sexuality and sexual relationships, as well as the possibility of having pleasurable and safe sexual experiences, free of coercion, discrimination and violence. For sexual health to be attained and maintained, the sexual rights of all persons must be respected, protected and fulfilled.” (WHO, 2006)

There has been an increasing shift towards holistic definitions of sexual health, rather than a limited focus on sexually transmitted infections (STIs), blood borne viruses and unplanned pregnancies. The most recent National Survey of Sexual Attitudes and Lifestyles (Natsal-3) survey, conducted across Britain with 15,162 people age 16–74 years found 1 in 10 women and 1 in 71 men reported an experience of ‘non-volitional’ sex (i.e., sex against their will) (Macdowall et al., 2013). Physical, psychological and sexual abuse is associated with sexual health outcomes such as sexually transmitted infections (STIs), unwanted pregnancies and sexual dysfunction (Coker, 2007, de Visser et al., 2014, Ellsberg et al., 2008, Garcia-Moreno et al., 2005, McMillan, 2013, World Health Organisation, 2013), so it is right that the WHO offer a broader holistic definition. Such a broad definition has been taken up in policy frameworks, including the Scottish *Sexual Health and Blood Borne Virus Framework 2015–2020,* developed to promote key outcomes including in relation to STIs and unintended pregnancies, inequalities, and sexual violence (Scottish Government, 2015).

In this article, one of a series of articles from our ‘DeMaSH’ study (Deprivation, Masculinities and Sexual Health), we first draw upon a social determinants of health framework as it intersects with our analysis of how masculinities in places influence sexual health and wellbeing. Here we draw out the importance of neighbourhood and community level factors, citing examples of how we might engage in interruptions in ecological systems (Hawe et al., 2004); elsewhere we focus on findings relating to holistic sexual health understandings, the blaming of women for sexual violence, and alcohol and sexual consent understandings. Thus, in this paper we only mention such findings tangentially. Here we prioritise narratives of violence because they often receive less emphasis than ‘bugs and babies’ within the sexual health field, and they provide a useful vehicle through which to convey the social embeddedness of behaviours, contextualised within environments of strain and adversity.

### 1.1 Levels of influence on sexual health and wellbeing, and the importance of neighbourhoods and communities

Immediate determinants of sexual health and wellbeing include, for example, individuals’ knowledge of sexual risks; however, although knowledge improvement is important for behaviour change, it is insufficient on its own to effect significant change as influences upon sexual health stem from factors beyond individual knowledge. At the more distal level, poverty is a significant contributor to various forms of gender-based violence (Jewkes, 2002). Epidemiological data reveal the impact of low socio-economic status (SES) upon sexual health (Arnold et al., 2011, Denning et al., 2011); this is compounded by those in low SES often being part of sexual networks with high underlying rates of STI's and HIV (Denning et al., 2011). The provision of laws (and law enforcement) to protect people from discrimination, violence and poverty can significantly improve the success of individual behaviour change strategies (Coates et al., 2008). For example, the funding associated with the 1994 US Violence Against Women Act resulted in significant effects on sexually violent behaviour (DeGue et al., 2014). By challenging dominant norms, change can occur that results in improved gender equity and reductions in sexual risks, violence and coercion. However, structural factors that can influence sexual health and wellbeing (e.g., poverty) tend to go beyond specific domains of health (e.g., HIV prevention), and tackling such issues are commonly for governments to implement across policy fields; as such, interventions to improve sexual health and wellbeing more commonly operate at community- or individual-level.

Given the geographical variations in sexual behaviours and HIV risks and acquisition (Wadsworth et al., 1996), what are the ‘chains of causation that might link place of residence with health outcomes’ (Macintyre et al., 2002). The ‘broken windows’ theory applied to STIs found deteriorated physical conditions of local neighbourhoods were associated with gonorrhoea rates, independent of poverty (Cohen et al., 2000). The acquisition of STIs has been associated with exposure to neighbourhood poverty during adolescence (Ford and Browning, 2014). Data from the US National Longitudinal Study of Adolescent Health also found neighbourhood influences upon earlier sexual initiation (Cubbin et al., 2005). Exposure to community violence has been associated with increased sexual risk behaviours (Cooper et al., 2015, Senn et al., 2016, Voisin et al., 2014). Communities in which violence in the family is acceptable experience increased likelihood of such violence (Pinchevsky and Wright, 2012). These studies are examples from an evidence-base that has begun to point strongly towards the association between community violence, peer acceptance of norms as well as acceptance of certain sexual behaviours with sexual health and wellbeing outcomes within communities. Indeed, a systematic review of the relationships between neighbourhood characteristics and ‘intimate partner violence’ (a common term used in the USA to refer to what more commonly referred to in the UK as domestic abuse), found ‘ample evidence to indicate that some aspects of neighbourhood may be risk markers or risk factors for IPV’ (Beyer et al., 2013, p. 41). However, other systematic reviews, examining risk and protective factors for sexual violence, have concluded there is little evidence on how community level factors are associated with sexual violence (DeGue et al., 2014, Tharp et al., 2013), and have noted that there are no included studies from Europe, and all studies are cross-sectional, highlighting an important gap in evidence. We would argue that qualitative work is needed to begin to bridge this gap and illuminate experiences *in* places (Popay et al., 2003), particularly experience of sexual health in relation to masculinity constructions.

### 1.2 Masculinities and sexual health

We sought to explore masculinity constructions within and across areas of high deprivation, in order to focus on local gender dynamics and the importance of experiences *in* places (Manzo, 2005), for the way these influence sexual health understandings and behaviours.

Connell defines ‘hegemonic masculinity’ as ‘the configuration of gender practice which embodies the currently accepted answer to the problem of the legitimacy of patriarchy, which guarantees (or is taken to guarantee) the dominant position of men and the subordination of women’ (Connell, 1995, p. 77). For a brief theoretical overview of hegemonic masculinity, and its relationship with other masculinities (e.g., protest masculinity, hypermasculinity) see Jewkes et al., 2015a, Jewkes et al., 2015b, or for more detail see Connell (1995). Here, we emphasise three points, which are particularly pertinent to our study: firstly, masculinity is embodied, structurally positioned *and* ‘performed’ (Archer and Yamashita, 2003); secondly, masculinities are relational – hegemonic masculinity is ‘a particular form of masculinity in hierarchical relation to a certain form of femininity and to nonhegemonic masculinities’ (Connell and Messerschmidt, 2005), and thirdly; gender does not operate on its own but in relation to other social dynamics such as class, race and sexuality (Connell and Messerschmidt, 2005). As Courtenay has stated, the ‘social structuring of ethnicity, sexuality and class is intimately and systematically related to the social structuring of gender and power (Courtenay, 2009). Thus, efforts to improve sexual health and wellbeing should be premised upon the understanding of gender as ‘a way of structuring social practice’ and ‘unavoidably involved with other social structures’ (Connell, 1995, p. 75).

Berg and Longhurst's review, ‘Placing Masculinities and Geography’, provides an excellent overview of the masculinities and geography research from its beginnings, so we opt not to rehash that here (Berg and Longhurst, 2003). We do draw attention to the lack of studies that bring together a focus on masculinities, place and sexual health. So on the one hand, spatial studies have explored relationships between area- and individual-level risks and individual HIV status (Feldacker et al., 2010); how the built environment influences young people's sexual risk behaviours (Burns and Snow, 2012); and where and how to place STI screening services (Balfe et al., 2010, Goldenberg et al., 2008). On the other, studies have focused on masculinities but not sexual health, such as those exploring rural and urban influences on masculinities and gender practices (Bye, 2009, Lysaght, 2002, Ni Laoire C and Fielding, 2006). Lysaght's study, for example, revealed the performative character of dominant and subordinate masculinities in Belfast, focusing on the way that spatial context affects the performance of gender identities (Lysaght, 2002). A scoping review (McDaid et al., 2012) underpinning our research, identified specific research gaps relating to intervention studies with adult heterosexual men from deprived areas.

Causal pathways link structural factors and sexual health and wellbeing outcomes; hence, for example, tackling gender inequalities can improve equitable interpersonal relationships (Taukobong et al., 2016) or reduce sexual risk behaviours (Gupta et al., 2008). To develop effective interventions, particularly those designed to improve gender relations so as to impact on sexual health outcomes, we require further research to identify ‘causal or contextual factors that are malleable and have greatest scope for change’, as well as the level at which intervening is possible within existing systems (Wight et al., 2016, p. 2).

## 2 Methods

Individual semi-structured interviews and focus group discussions were conducted with men and women living within the same geographical localities, as described in more detail below. We anticipated that men, and women, might be more willing to talk about some sensitive issues, or personal experiences (e.g., experiences of domestic abuse, sexual consent negotiation, sexual histories etc.), in individual interviews rather than in a group context. By contrast, as we anticipated that the ways in which understandings of both sexual health and socially appropriate performances of masculinity and femininity are co-created in interaction with others, we considered focus group discussions to be more appropriate to explore negotiations of such meanings between participants. We chose to conduct the focus groups as single sex discussions. Since our primary interest was in the interrelationships between constructions of *masculinities* and sexual health, attitudes and behaviours, we first conducted focus groups with groups of men who were friends or who knew each other, so that we could capture men's ‘performances’ of masculinities in the presence of other men (De Visser et al., 2009, O'Brien et al., 2005, O’Brien et al., 2009). As sexual behaviours and attitudes are often related to peer influence, we anticipated that groups of friends may share some similar views, attitudes and behaviours, or be comfortable talking about some differences in this context (Hyde et al., 2005). The all-male focus groups were conducted in parallel with individual interviews with men (Phase 1 data collection). Towards the end of this phase we began to recruit women from the same localities to all-female focus groups as well as to individual interviews (Phase 2 data collection). In practice, the data were collected semi-sequentially, as there was a small temporal overlap between phases 1 and 2. Men's accounts were presented back to women (in both individual interviews and focus groups) in anonymised form, mostly in terms of generalisations (e.g., “Some men have told us that …what do you think of that?”). Our strategy was to present an alternative to the women's views, for example if they offered a negative view of men we presented a positive comment a man or men had made to facilitate deeper exploration of women's experiences.

### 2.1 Participants

We recruited men and women, aged 18–40 years, who resided in areas classified as the most deprived. Participants lived in major cities within Scotland (Glasgow, Edinburgh, Dundee) or rural/semi-rural settings in the Highlands. We selected these locations based on a combination of sexual health outcomes (e.g., rates of unintended pregnancies) and the degree of urbanicity/rurality, as assessed by the Scottish Government's 6-fold Urban/Rural Classification, 2011–2012 (Scottish Government). We then focused on small areas classed as within the most deprived deciles of deprivation within Scotland, using the Scottish Index of Multiple Deprivation (SIMD). At the time of interview, all participants resided in a SIMD 1 area, i.e., within areas classed as the 10% most deprived.

Sexual risk-taking and sexual safety are constituted in social relations of heterosexuality (Holland et al., 1998). As such, we felt it essential to explore how *women* in these communities view men's accounts. The exclusion of women's perspectives would neglect the interplay between women's and men's perceptions, expectations and experiences of masculinities, and the ways in which men ‘perform’ their gender with sexual partners, within and beyond the purview of other men. However, we fully acknowledge that masculinities act in relation to each other, and how men relate to other men is also fundamentally important.

### 2.2 Recruitment

We adopted a multi-faceted recruitment strategy, which included: passive approaches such as flyers, postcards (which included a QR code and details of how people could find out more about the study by texting or visiting the study website); and, more proactive approaches, such as talks in community settings, approaching potential participants directly in pubs and community settings, and engaging gatekeepers within community organisations. The proactive approaches required time to build trust and rapport, but proved the most effective means of recruitment. These early interactions also provided valuable insights into the areas and communities in which participants lived. Recruitment occurred between February 2014 and April 2015. Interviews lasted between 45 min and 2 h (1 h on average) and focus groups between 1 and 2 h (1.5 h on average). Each participant received a £20 gift voucher as a gesture of thanks for their time.

### 2.3 Developing the topic guide

In this study we embraced a holistic definition of sexual health, which encompasses disease prevention but also positive relationships free of coercion and violence (WHO, 2006). Our pilot work focused on developing the topic guides for focus groups and individual interviews. Following earlier research on men and masculinities (De Visser et al., 2009, Gill et al., 2005), we anticipated that the use of images could facilitate discussions around social constructions of masculinities and femininities; the pilot stage afforded a valuable opportunity to test and refine the selection of images (see Appendix A). We experimented with a variety of images and settled on a set of images of 6 men and 6 women, as well as carefully selected images to encourage discussions of gender-based violence. This latter set included images of: 1) an unconscious woman being carried by three young men, allegedly after her drink had been ‘spiked’ (using Rohypnol, a ‘date rape’ drug); 2) an image from a Police Scotland campaign depicting a rugby player with the text ‘I’m the kind of guy who doesn’t have sex with a girl when she's too drunk. Are you?’; 3) three still images from a television campaign advert about rape within the context of a relationship; 4) a woman cowering, with bodily bruises evident, and a man standing over her clenching his fist, with the accompanying text ‘Women deserve equal rights….and lefts’ and 5) AWARE Centre campaign image (a fist emerging from a man's mouth grabbing a woman's hair) with accompanying text ‘Verbal abuse can be just as horrific’. These images were chosen to reflect a range of gender based violence, to explore whether, and how, these images were perceived as gender based violence by participants, and to prompt discussion of what can be a very difficult topic. The images also reflected the fact that across the spectrum of violence, men predominate as perpetrators (Fleming et al., 2015, Macdowall et al., 2013, World Health Organisation, 2013), but this is not saying all men are violent. The images were also selected to reflect the capacity for men to make positive choices to not engage in sexually violent practices.

### 2.4 Interviews and focus groups discussion topics

We began by asking participants about their experiences of living/growing up in the area (‘*what was it like growing up in* [area]?’), guiding respondents towards discussions that enabled us to explore the ways in which ontological identity (sense of self) and biographies are bound up with the places in which people live (Popay et al., 2003). We sought to understand influences on masculinity and gender practice formation *in these specific localities*, characterised, as described above, by high levels of social deprivation. SR conducted interviews and focus groups, with another researcher present during focus groups taking notes [KL]. This was followed by questions on their views towards men and women (using the 12 images noted in 2.3 above), probing for similarities and differences to people in their own area when growing up and also at present. The five images depicting gender-based violence were then used to prompt discussions on their understandings of sexual consent as well as views towards, and experiences of, physical, sexual and verbal abuse. Participants were then asked about their sexual health understandings, knowledge and behaviours (e.g., ‘how did you learn about sex?’, ‘what does sex mean to you?’, views towards condoms).

### 2.5 Analysis

Interviews and focus group discussions were transcribed and annotated to link, where possible, to our observations of relevant non-verbal communication. Transcripts were analysed using the Framework Approach (Ritchie and Lewis, 2003), focusing both on *a priori* areas of interest drawn from the topic guide whilst also allowing for unanticipated themes to be systematically identified inductively (Pope et al., 2000). Using QSR NVivo software we employed an iterative approach to our thematic analysis: a selection of the transcripts was read independently and repeatedly by two research team members to generate an initial coding framework; other team members independently reviewed this in light of their own readings of selected transcripts related to their areas of expertise. The coding framework was then further refined through extensive discussion between the whole team, before all data were coded. Data were then indexed, charted systematically and organised using matrices in which each participant is represented by a row and each theme by a column. Constant comparison was carried out to ensure the analysis represented all perspectives, including any ‘deviant’ cases and to avoid individual or small group researcher bias. Throughout we have used pseudonyms and note the type of interview with Int (interview) or FG (focus group) in parentheses, along with the participants’ age at interview.

### 2.6 Ethics

Ethical approval was given by Glasgow Caledonian University, School of Health and Life Sciences research ethics committee (REF: SYEC12/APP202).

## 3 Findings

We recruited 116 people aged 18–40 years to the study: 68 men (mean age 30) and 48 women (mean age 29) (see Table 1). We conducted 35 individual interviews (19 men, 16 women) and another 81 took part in a total of 18 focus group discussions (11 with men and 7 with women); no individual took part in both. Most participants (see Table 2) were: unemployed, living in social housing, with at most school level qualifications (around one quarter had no qualifications); and married /currently in a long-term relationship (43%) or single (49%) (a few described themselves as divorced/separated or in an open relationship).

**Table 1:** Number of interviews and focus groups conducted, by recruitment area and gender.

		**Recruitment area**			
	**Total**	**Glasgow**	**Edinburgh**	**Dundee**	**Highlands**
**Focus groups**	**18**	**5**	**5**	**4**	**4**
Men	11	3	3	3	2
Women	7	2	2	1	2
**Individual interviews**	**35**	**9**	**9**	**8**	**9**
Men	19	5	4	5	5
Women	16	4	5	3	4

**Table 2:** Demographic data for interview and focus group participants, by gender.

	**Total, n (%)**	**Men, n**^a^	**Women, n**
**Age, years**			
18–25	35 (30)	21	14
26–30	32 (28)	17	15
31–35	19 (16)	9	10
36–40	30 (26)	21	9
**Relationship status**			
**(**a**1 missing)**			
Long-term relationship/married	50 (43)	24	26
Single	56 (49)	36	20
Separated/Divorced	6 (5)	4	2
Open relationship	3 (2)	3	–
**Highest educational qualification**			
**(**a**1 missing)**			
None	25 (22)	14	11
Standard grades	51 (44)	31	20
Vocational/apprenticeship	3 (2)	3	–
Scottish Highers/college qualification (e.g., NC, HNC)	22 (19)	11	11
Undergraduate degree	3 (2)	3	–
Prefer not to say	10 (9)	4	6
**Employment status**			
**(**a**1 missing)**			
Unemployed	71 (62)	45	26
Part-time	20 (17)	6	14
Full-time	19 (16)	14	5
Prefer not to say	5 (4)	2	3
**Housing situation**			
**(**a**9 missing)**			
No fixed address	7 (6)	6	1
Living rent free (parents, friends)	19 (18)	13	6
Renting/LAH	75 (70)	39	36
Owner occupier	5 (4)	5	0

% rounded.

a Missing data for some participants so numbers do not always add to 116(total).

### 3.1 Neighbourhood/community: environments of strain and adversity

As per our inclusion criteria, all participants were resident in an area of high deprivation, but most also grew-up in the same or a similar area. Childhood experiences, and many of the accounts of contemporary life, were thick descriptions of poverty, material deprivation and hostile environments. In addition to examples of experiencing a lack of basic nourishment and money, many also described how manifestations of poverty affected the way people looked. For example, Angela said

…probably the biggest thing is the look of people. Kinda people in [area] kinda look pretty grey, pretty thin, pretty miserable looking to be honest. (Angela, age 34, Glasgow, Int3).

The language many men and women used to describe their areas was, at times, stark (‘hellhole’,’ shithole’, ‘rough’), mirroring findings from other work, and sometimes even the exact same terms (Deuchar, 2010). People, and more often women, invoked strong emotional responses when recalling the youth violence in these areas (e.g., feeling ‘petrified’), although many offered accounts of change, such as reductions in gang violence or drug misuse problems, in their areas.

Yeah there was a lot o’ gang fights back then, yeah. There was a lot o’ like from scheme [housing estate] to scheme. It's not like that anymore but yeah, each scheme used to fight, have a day that they went up and fought each other, sort of thing. There was a lot o’ gang violence back then. (Emma, age 32, Dundee, Int1)

Overall, what emerged strongly in relation to accounts of manifestations of poverty within places was people's habituation to them, often expressed through shrugs of shoulders and a neutral tone of voice. Angela (age 34, Glasgow, Int3) talked of growing up with poverty but “you just kinda deal with it”. Across the many descriptions of drug misuse, violence and criminality they experienced or were aware of in their locality, we failed to read such habituation as dispassion. Of course, not all participants who resided in these areas of high deprivation necessarily lived in poverty themselves. Nevertheless, we were struck by the shadow cast by poverty, drugs and violence, which appeared never to be far from the doorsteps of almost all participants, and for many it was an intrinsic part of negotiating everyday life. Hence, the stresses and strain of residing in some challenging environments varied across accounts but appeared present in some form.

Participants’ accounts often noted the influence of wider structural level influences bearing down on them. Luke, for example, pointed out that individuals’ alcohol consumption should not be detached from the influence of poverty

L: Well, I think… in most non-affluent areas it's like that. It's just people get desperate, don't they? And then they get drunk tae try an' hide their desperations and their reality sort of.

Int: So when you say they get desperate, how do you mean?

L: Well, you see… now you see all these people going tae food banks an' stuff like that 'cause they cannae feed their kids or they've no' got a job so they sit aboot the hoose all day bored so they just go 'Ah, fuck it, I'll get drunk' (Luke, age 21, Edinburgh, Int3)

Daniel spoke with passionate concern about Dundee's level of mortality related to drug misuse but added

building a’ that [new cultural facilities] on the waterfront [in Dundee]. What's that for? It's no’ for us. That's no’ for us. It might benefit the city in general wi’ tourists and things, but when you come out o’ Dundee you’ll see it, it's a nice place, but behind that it's still Dundee. It's still the shithole that's here. (Daniel, age 38, Dundee, Int2)

Thomas challenged the notion that individuals can easily change their behaviour:

people just don't realise what it's like tae live and kind o' grow up in some o' these places and I think that they're kind o' ignorant when they think that they can just change a couple o' things and it'll make everything awright (Thomas, age 32, Glasgow, Int5)

The breeding of alienation was evident in the class injury experienced by many respondents: the way in which class narratives were woven through narratives of structural disadvantage highlights the way in which masculinities were, for some, constructed in relation to class identity. Ally, for example, interwove his politics throughout his interview, from challenging housing regeneration to immigration:

I feel like a lot o' this immigration is a way tae drive doon wage costs, you know what I mean? Rather than any great plan tae, you know, multiculturalise Britain, … No negative effect on doctors or middle class people, you know, getting their cheap coffee or, you know, their nanny or whatever it is…(Ally, age 40, Glasgow Int1).

There was a tendency for men's recalled practices and beliefs to point towards a traditional hegemonic masculinity structuring their gender relations. However, at the intersection of gender, poverty and class (i.e., how these systems overlap), we read corporeal performances – sexual behaviours and violence – as reflecting responses to impoverished situations by both adhering to and contesting this traditional hegemonic masculinity. Connell has described a ‘Protest masculinity [which] is a marginalised masculinity, which picks up themes of hegemonic masculinity in the society at large but reworks them in a context of poverty' (Connell, 1995, p. 114). The redemption scripts some men offered conveyed a disruption and contestation of hegemonic masculinity, but not in line with protest masculinity. Through abstaining from youth violence or embracing a monogamous long-term relationship rather than multiple sexual partners, these ‘changed men’ challenged the hegemonic gender norms they had absorbed, even if not disrupting them completely. This focuses our attention on gender as ‘contextually bound and responsive to changing circumstances and relations’ (Doull et al., 2013, p. 340).

### 3.2 Violent milieux

A particularly prominent account of ‘place’ – commonly associated with growing up, but also peppering accounts of contemporary life – emphasised the violent milieus, in which a narrow range of performances of masculinity felt possible. Two particular types of violence narratives emerged across our data: youth violence and domestic abuse. The former was not a specific question in our topic guide whereas the latter was, via the images we used (see 2.4).

#### 3.2.1 Youth violence

Men who discussed their experiences of engaging in youth violence –commonly, but not exclusively, our Glasgow participants – gave a variety of reasons for this, from boredom to the acquisition of status, mirroring others’ findings (Deuchar, 2010, Deuchar and Holligan, 2010). In these narratives, we read a mixture of choice (“Just dinnae [didn’t] feel coerced intae [into] it, just actively joined in”) and obligation (often even at different points in the same interview), suggesting that their violent behaviours were unavoidable and normalised in their community.

gang fights, just fae [from] where you’re fae – different side o’ the street… everybody else was daen [doing] it, but it was the only thing there was to dae at the time (Jim, age 22, Glasgow Int2)

Peer group networks are a key site for the construction and (re)production of masculinity: young men engage in specific types of (hetero) sexual practice in order to gain social approval from peers (Richardson, 2010). For many men in this study, a collective masculinity centered on youth violence, appeared crucial for their embodied heteromasculinity. The ‘doing’ of gender was protective, chivalrous and potentially violent:

it would be forty, fifty o' us boys hanging aboot a street at night so there'd maybe be four or five girls hanging aboot with us. But if anybody had came doon and tried tae fight with any o' the girls and they had a whole big, massive squad o' us tae fight (Thomas, age 32, Glasgow Int5)

Such performances of violence, as a response to their environment, can be ways to gain power, and so demonstrate gains from adherence to hegemonic masculinity. However, such behaviours are more commonly deemed a ‘protest’ masculinity whereby displays of hyper-masculine behaviours draw upon the hegemonic ideal but fail to reap the full rewards of it and certainly it is not a contestation of hegemony (Connell, 1995). That said, we also noted men sought emotional support in these collectives; thus, agency enhancement was sought through non-performative aspects. Neither were these fixed practices, as the desistance literature has made clear in relation to ‘growing out’ of crime (Maruna, 1997, McNeill et al., 2012), even within circumstances of continuing structural constraints. Each of these men conveyed a trigger for the break: facing jail time (Luke); gaining employment in a middle-class environment, lending itself to desistance from gang violence and embracing new hobbies (Ally); desire for a long-term relationship (Billy); becoming a father (Thomas). This underscores masculinities as dynamic, rather than fixed (Dworkin et al., 2015).

Women's accounts also conveyed the negotiation of such violent milieus, sometimes evoking strong emotional responses (feeling ‘petrified’), and likened gang violence to a ‘war zone’. However, what stood out across the women's data was their perceptions of high social cohesion in their very localised community, although this tended to be in sororities, with female neighbours and other women to whom they were not related (e.g., nursery/school staff, other mothers). In such relational accounts, we discerned the living of almost separate lives by women and men across these communities, impacting on how the sexes understood, and communicated with, each other.

#### 3.2.2 Domestic abuse and sexual violence

Across interviews and focus groups, it was clear that men's and women's biographies commonly conveyed gender practices rooted in a domestically violent milieu. We heard men talk about domestic abuse as a common feature of their communities, or personally witnessing domestic abuse. Here Ally talks about it within his community

It's just something that I've seen for years, aye [yes]. It's a common thing, aye. You know? You might no' see the physical acts o' violence. You dae [do] sometimes. But you see the way women are. (Ally, age 40, Glasgow Int1)

Those who spoke of more personal experiences, including witnessing domestic abuse as a child, mostly described certain incidents.

I grew up wae [with] my mum, domestic violence, seen it when I was a child and I don’t like it. I remember being stuck in a room, my bedroom, and I could hear my mum getting battered off all the walls (Ryan, age 36, Dundee Int4)

Another man recalled an extreme episode of violence perpetrated on a woman he had been in a relationship with by her ex-partner, which resulted in him later being diagnosed with PTSD [we provide no further details here to preclude deductive disclosure of either party's identity]. One man spoke of an aunt being subject to what has been referred to as ‘intimate terrorism’ – the sustained use of physical and other tactics to exert control (Stark et al., 2013). During these discussions, we witnessed some men's clear upset when recalling such incidents, particularly when recounting witnessing domestic abuse towards their mothers. For example, in one focus group a man had tears in his eyes, and in another a man's tone of voice contained anger, which was not present when discussing other issues throughout the focus group. Men who had witnessed domestic violence as a child, and/or in later life towards sisters or other female relations, were somewhat more likely to be empathetic towards women and stauncher in their condemnation of such violence. Despite the commonality of witnessing domestic abuse, and perceiving it as prevalent within their communities, most men conveyed a strong view against such violence, often in descriptions of what was acceptable for a man to do (invoking the ‘real man’ trope) (Salter, 2015) – the appropriate etiquette for ‘real’ men.

I just think men like that [who domestically abuse] are bullies, cowards. Total cowards. That they have tae hide behind doors and tell lies and, aye, it's disgusting, tae be honest wi' you. And that's a' [all] part o' the way that we grew up as well – if you were gonnae fight wi' somebody then you had a fight wi' them. You stood oot on the street and the two o' youse fought wi' each other like men “Thomas, age 32, Glasgow Int5”.

Thus, in contrast to the youth violence that many felt was normalised and in which some revealed they partook, far more men rejected the practice of violence towards a woman. Despite this, it remains prevalent.

Across interviews and focus groups, the image we used to facilitate discussions of domestic abuse (Image 4) commonly resulted in men expressing wholehearted agreement that women had a right to be treated equally, without the spectre of violence:

At first I just seen ‘women deserve equal rights’, like yeah, yeah women deserve equal rights, but then I seen ‘and lefts.’ A woman doesnae deserve to be beaten "Scott, age 40, Dundee Int5".

However, at times, humour was wielded, in ways that minimized the seriousness of the issue, particularly during focus group discussions, as in this discussion of image 4 (domestic abuse), in which the image of a bruised and cowering woman led to claims of her being a ‘golddigger’:

P2: You can see the pain in her face, you know what I mean?

P1: Or it could be that, eh? But then I think she’ll end up selling the book later on, sorta thing, like, telling her story.

(Laughing)

P1: No, honestly. I’m no’ being funny about it, I’m no’, honestly. It just seems like that kinda thing. (Dundee FG2, Men)

The group context could have increased embarrassment such that humour may have been used as a way to cover discomfort. However, these were not one-off jokes, smirks, sniggers or laughs. When challenged to explain their laughter, smiles or smirks, a level of back-tracking took place and discussions often, but not always, moved towards a more condoning tone. Men may have been performing in front of each other, or occasionally felt awkward in front of a female researcher, but they were not awkward when stating with certainty that many women ‘cry rape’.

Well, you don’t want tae wake up the next day and then the lassie's like that, ‘What happened last night? Did we have sex? Blah, blah, blah.’ And then she goes away and says ‘I got raped last night.’ Know what I mean? ‘Cause she's too drunk to remember. (Dundee FG2, Men)

Some women also offered similar views, such as Denise (age 32, Edinburgh, Int3): “if she's too drunk she doesnae know what she's doing and in the morning she could forget and obviously wake up next tae him, cry rape”.

The blaming of women for incidents of sexual violence was salient in the data. Irrespective of whether the assignment of blame was nuanced with some ambiguity, or categorically stated from the outset, the caveats and considerations that followed almost always led back to attribution of blame to women. This was particularly marked in men's discussion, while women were more likely to attribute some blame to men. It may be that the women's greater levels of personal experience of gender-based violence affected responses. Indeed, we heard horrific stories of abuse involving stabbings, brutal beatings and sustained coercive control.

I was in a relationship where I suffered domestic violence, I put up with it for 4 years and I was ashamed. I felt so ashamed, I couldn’t tell anybody. So I’d need to stay in and I’d need to put foundation over my bruises. And it wasn’t just physical abuse, it was a mental abuse. He made me feel like I was worth, worth nothing. Sometimes I wanted to kill myself it was that bad. (Jodie, age 29, Dundee Int2)

Jade (age 36, Dundee Int3), viewed image 3 and said

If I had a’ known, years ago, that you can actually get a boyfriend done with rape, then I would have done it – it's as simple as that. But obviously, it's hard to establish. You’re in a relationship; you’ve got a child to the person.

Even when presented with some of the more positive views expressed by the men, many women expressed doubts about their veracity, sincerity and honesty, and denied that they were reflective of their own experiences of men in their areas.

I would actually say that if the girl doesnae fit the bill, men, boys, are, they are cruel. They are. I mean, you'll get… they'll call them names or they'll be nasty tae them. Yeah, I think that. It's just the way the world is in society. (Shannon, age 33, Edinburgh, Int1).

Of the sixteen women we interviewed, thirteen revealed that they had witnessed and/or been subject to personal experiences of domestic abuse. It is perhaps not unsurprising given such biographies of violence, and negotiating their femininity in relation to such violence, that women held such views towards men. Almost all of these comments were made with a serious tone of voice, with the exception of one focus group in which the women collectively laughed at a man's positive statement read to them. Nevertheless, many wanted such sentiments from men to be true. However, it is also important to note that we also heard women refer to other women as whores, tarts and bitches, and some also blamed victims of sexual violence, thus using language that supports inequalities and the gender order.

### 3.3 Intrapersonal: family structures

Creighton and Oliffe recognise the importance of men's peer, partner and parental relationships (Creighton and Oliffe, 2010). Most of the men in this study did not grow up within a conventional nuclear family. Ryan (age 36, Dundee Int4) and Rab (age 23, Highland Int2), for example, recalled growing up in and out of the care system. Many others did not grow up with their biological fathers present in their lives, but instead were parented by a mother or other female relative (e.g., grandmother), such as Connor (age 20, Dundee, Int1). Thus, for many of the men, the primary role model for their developing gender identity and practice was not a biological father, with women or even the state being a key influence. Littered through the childhood stories were substance use issues (alcoholic mother or violent drunken father), violence – particularly domestic abuse – and fragmented relationships. As previously stated, the emotional connection sought in certain masculine collectives was perhaps for some a way to gain a sense of family.

However, we discerned no clear line between family structures and egalitarian/non-egalitarian views. Similarly, a weak association has been found between ‘father absence’ and physical and verbal aggression (Boothroyd and Cross, 2017). Five male interviewees seemed utterly lacking in empathy for women, as was particularly prominent during the discussions using images of abuse. These men offered consistently non-egalitarian views resting on a strong gender division of labour, and believed a positive relationship was, simply, one in which women were not hit. We sought to determine whether these men's biographies differed from men we deemed ‘mixed-egalitarian’ (who offered views mostly consistent with gender equality). Most of the non-egalitarians shared a biography of chaotic family structures, such as Ryan and Rab raised in and out of the care system. Ryan, an interviewee with a profound lack of empathy for women (despite witnessing domestic abuse as a child), seemed bereft of aspiration for a positive romantic relationship, almost wary of expecting much, but elsewhere he was animated in his aspiration for employment: ‘clean-cut, suit, tie, probably a good job, nice car’. Yet we also interviewed Connor (age 20, Dundee, Int1), who was raised by his grandmother from age 5. He offered two passionate and engaged responses when discussing women in his life and his thoughts and aspirations for a positive relationship.

Connor: ‘You don’t want somebody that's gonnae be cleaning hooses and that, no, you want somebody that's independent as well…You don’t want them tae just come hame, cook, clean, and give the bairn [child] their dinners…’

Interviewer: So why not?

Connor: ‘Cause it's maybe a family if people dae things together.

## 4 Discussion

Our study focused on masculinity constructions within and across areas of high deprivation, to focus on the importance of experience *in* places. Masculinities originating from strain and adversity have been explored both theoretically and empirically (Connell, 1995, Jewkes et al., 2015b). However, as a scoping review found a significant gap on work that brings together masculinities, deprivation and sexual health, with adult heterosexual men (McDaid et al., 2012), we believe this is the first UK study operating at this intersection. As has been made clear by others (Berg and Longhurst, 2003, Connell, 1995, Messerschmidt, 2012a), and we reiterate, it is important not to decouple masculinities from their social context. However, causal pathways in relation to complex problems are often diverse and interwoven (Wight et al., 2016). As such, a challenge within this study was to identify such pathways given the confluence of factors such as social gender norms, peer cultures and early years’ experiences of violence (Jewkes et al., 2015b).

If we return to the WHO definition of sexual health, we are reminded of its holistic nature, where disease prevention sits alongside positive, respectful and pleasurable relationships, free of coercion, discrimination and violence (WHO, 2006). Masculinities are directly and indirectly associated with risks for STIs and HIV, as well as with various forms of violence (Brown et al., 2005, Fiaveh et al., 2015, Fleming et al., 2015). Whilst a range of masculinities may theoretically be available to men in any social context, the men in this study constructed their gender in circumstances in which they had little access to economic resources. Some seemed to recall practicing a ‘protest masculinity’, whilst others reproduced aspects of hegemonic masculinity. It was clear that localised, socio-cultural influences did not appear to enable more egalitarian expressions of masculinity; indeed we experienced an undertone of simmering resentment towards women within much of the men's data, and at times a more ‘gentrified misogyny’ was evident (Glosswitch, 2016). We were struck by the level of blame attributed to women for incidents of sexual violence, which regardless of the extent of their discussions, with caveats and considerations, seemed always to lead to some level of blame upon women; although, some of the most aggressive views towards women emerged in relation to women who were deemed to have transgressed gender norms for ‘appropriate’ femininity in relation to sexuality and sex. Phipps suggests, ‘it is possible that the working-class woman may be more at risk of sexual violence as a punishment for unfeminine behaviour…’ (Phipps, 2009, p677). Men's and women's exposure to these localised, socio-cultural influences were delineating boundaries of acceptable social roles within romantic relationships. We are not suggesting here that women lack agency, but we note again that 13 of our 16 women interviewees recalled directly witnessing and/or being a survivor of domestic abuse. These experiences of power convey a social order, and can act to silence: silencing of men's desires for intimacy and silencing of women's aspirations for more equitable and fulfilling relationships.

Given our respondents were age 18–40 years, we have focused on a portion of the lifecourse which has been under-researched (McDaid et al., 2012). Whilst we gathered similar narratives to those found in other work, around men's claims to power through sex and violence (Courtenay, 2000), we also heard how some men, when shifting towards desiring intimacy, were often devastatingly unprepared. The masculinity norms adopted and enacted throughout youth appeared to have a lasting negative impact on these men as they were aging. Such constrained masculinities work against positive gender relations, and for the achievement of even more positive and intimate relationships. We reiterate Connell's clear articulation that masculinities are not fixed (Connell, 1995), so contexts in which the practice of heteromasculinity requires particularly gender/sexual behaviour (Messerschmidt, 2012b) are subject to change. Indeed, across the ‘redemption scripts’ we heard we heard change occurring amidst the same set of more distal, upstream, determinants. However, such change tended to occur around significant life events, such as a threat of jail – a jolt rather than an evolution in attitudes and behaviours. For men to attain the positive, intimate relationships that some said they desire, the masculinity norms they embrace need to shift towards respect for gender equality. De Visser et al. (2009) found that aspects of ‘masculine capital’ can be traded to allow men to compensate for ‘non-masculine’ behaviours (De Visser et al., 2009). However, most of the men in this study lacked economic resources, and signifiers of masculinity they valued (e.g., car, being a ‘breadwinner’), so in order to adopt practices of masculinity that include making themselves emotionally vulnerable in an intimate relationship, what scope can they be offered in the trade of masculine capital whilst the dominant localised socio-cultural constructions of masculinity devalue intimacy and gender equality? As the ‘Stepping Stones’ intervention work shows, ‘men's improving livelihoods afforded men the opportunity to materially demonstrate the social changes – in the form of shifts in masculinity’ (Gibbs et al., 2015).

When such wider socio-structural forces work against positive and more egalitarian gender relations, and inhibit men acting on aspirations for intimate relationships, individual-level interventions alone will not bring about transformative change. Tackling gender inequalities can improve equitable interpersonal relationships (Taukobong et al., 2016) or reduce sexual risk behaviours (Gupta et al., 2008). Interventions are needed to tackle normative roles and assumptions regarding gender and masculinities that conflict with respect for equality, and cast collective agency as arising from shared and mutual experiences with structural features. Men's sexual risk behaviours have reduced through interventions which have addressed the negative impact of traditional gender norms (Hatcher et al., 2014, Jewkes et al., 2008, Solorzano et al., 2008). Such gender transformative interventions have sought to transform social norms, and systems that sustain gender inequality and violence (Fleming et al., 2014, Jewkes et al., 2015a). This and other research illustrates potential for change, but this is likely to lead only to incremental gains in the absence of measures to also tackle underlying structural constraints, such as poverty.

Our work has limitations. Having a female interviewer/moderator for the focus groups and interviews meant we were not able to investigate whether responses would have differed with a male interviewer. Our focus on relationality as between women and men – although we have not completely excluded relations between men – reflected our primary interest in sexual health among heterosexual adults in this study. However, our study is enhanced by the large sample size and plurality of voices, including women's, those from urban as well as semi-rural areas and age ranges. We also employed both focus groups and individual interviews, so whilst we observed ‘performance’ of gender in group discussions, for example sexist comments backed with laughter, we also noted similar, if a little less boisterous, attitudes and beliefs in individual interviews. We took several steps to ensure the credibility of this work: we adopted methods, questions and analysis that are widely accepted; we piloted our topic guide and the use of specific images to prompt discussion; we did not recruit from one setting nor via one type of approach; and specific ploys were utilized during questioning. We used iterative questioning and probes to elicit detailed data and remained alert to apparent contradictions which were followed-up. When men or women offered a negative view, we offered a vignette of a more positive perspective, and vice versa (conveying a negative/positive view as credible and creating space for people to revise their view) to allow us to probe participants’ understandings more fully. Whether men expressed more egalitarian views and rejected all violence towards women, for example, in order to present a socially acceptable masculinity to a female researcher is questionable given only one man of 68 in the study offered consistently egalitarian views. The ways in which women expressed their scepticism about the veracity or sincerity of men's more positive comments seems wholly consistent with the accounts of sometimes sustained and horrific abuse that peppered life stories. In this sense, it is not surprising that many women responded with a smirk or a disbelieving ‘aye, right’. Indeed, our own experience of conducting the fieldwork included interactions that indicated an acceptance and normalisation of problematic attitudes and behaviours.

## 5 Conclusions

Drawing upon a social determinants of health framework, we found a complex picture of multi-level influences upon masculinity construction across the life course: gender norms within contexts of poverty are reinforced at a local level to create a gendered environment, which is then taken on in individual behaviours and attitudes. The accounts revealed through this research highlight the depth of the challenge in improving understandings and practices of sexual health, tackling gender norms and helping foster more positive and equal relationships in these contexts. Localised, socio-cultural influences did not appear to enable more egalitarian expressions of masculinity, and were setting out boundaries of acceptable social roles within romantic relationships. For men to attain the positive, intimate relationships that some said they desire, the masculinity norms they embrace need to shift towards respect for gender equality. We strongly recommend interventions occur across all levels of influence, drawing upon evidence from multi-disciplinary and multi-agency approaches, due to the limits of what individual-level interventions can achieve. However, the contexts our participants recalled and described are the real-world ones that policies need to work within; therefore, the importance of experiences *in places* needs to be better understood if sexual health and wellbeing interventions are to achieve more than incremental gains.

## Appendix A Images used in individual interviews and focus group discussions

Images of men

Images of women

Images used to facilitate discussions on sexual consent and domestic abuse

IMAGE A

IMAGE B

IMAGE C

IMAGE D

IMAGE E
